# Extensive Microbial Processing of Polysaccharides in the South Pacific Gyre via Selfish Uptake and Extracellular Hydrolysis

**DOI:** 10.3389/fmicb.2020.583158

**Published:** 2020-12-18

**Authors:** Greta Reintjes, Bernhard M. Fuchs, Rudolf Amann, Carol Arnosti

**Affiliations:** ^1^Department of Molecular Ecology, Max Planck Institute for Marine Microbiology, Bremen, Germany; ^2^Lethbridge Research and Development Centre, Agriculture and Agri-Food Canada, Lethbridge, AB, Canada; ^3^Department of Marine Sciences, University of North Carolina-Chapel Hill, Chapel Hill, NC, United States

**Keywords:** polysaccharides, substrate processing mechanisms, *Alteromonas*, dissolved organic matter, bacterial community composition, carbohydrates, extracellular enzymes, laminarin

## Abstract

Primary productivity occurs throughout the deep euphotic zone of the oligotrophic South Pacific Gyre (SPG), fueled largely by the regeneration of nutrients and thus recycling of organic matter. We investigated the heterotrophic capabilities of the SPG’s bacterial communities by examining their ability to process polysaccharides, an important component of marine organic matter. We focused on the initial step of organic matter degradation by measuring the activities of extracellular enzymes that hydrolyze six different polysaccharides to smaller sizes. This process can occur by two distinct mechanisms: “selfish uptake,” in which initial hydrolysis is coupled to transport of large polysaccharide fragments into the periplasmic space of bacteria, with little to no loss of hydrolysis products to the external environment, and “external hydrolysis,” in which low molecular weight (LMW) hydrolysis products are produced in the external environment. Given the oligotrophic nature of the SPG, we did not expect high enzymatic activity; however, we found that all six polysaccharides were hydrolyzed externally and taken up selfishly in the central SPG, observations that may be linked to a comparatively high abundance of diatoms at the depth and location sampled (75 m). At the edge of the gyre and close to the center of the gyre, four of six polysaccharides were externally hydrolyzed, and a lower fraction of the bacterial community showed selfish uptake. One polysaccharide (fucoidan) was selfishly taken up without measurable external hydrolysis at two stations. Additional incubations of central gyre water from depths of 1,250 and 2,800 m with laminarin (an abundant polysaccharide in the ocean) led to extreme growth of opportunistic bacteria (*Alteromonas)*, as tracked by cell counts and next generation sequencing of the bacterial communities. These *Alteromonas* appear to concurrently selfishly take up laminarin and release LMW hydrolysis products. Overall, extracellular enzyme activities in the SPG were similar to activities in non-oligotrophic regions, and a considerable fraction of the community was capable of selfish uptake at all three stations. A diverse set of bacteria responded to and are potentially important for the recycling of organic matter in the SPG.

## Introduction

Heterotrophic bacterial communities process marine organic matter in ocean waters, on particles, and in sediments, collectively cycling an estimated half of primary productivity (Azam, 1998). Intensive studies of bacterial communities have revealed patterns of bacterial community response to seasonal and spatial differences in primary productivity (e.g., Fuhrman et al., 2006; Gilbert et al., 2012; Landa et al., 2015; Teeling et al., 2016) in coastal and productive regions of the ocean. Within the past decade, investigations of spatial and temporal gradients in genomes, genes, and proteins have revealed finely tuned microbial responses to variations in environmental conditions and changes in phytoplankton productivity (Teeling et al., 2016; Mende et al., 2017). However, much of the total area of the ocean includes gyres that are characterized by lower variability in primary productivity and often lower nutrient concentrations, yielding communities adapted to more stable oligotrophic conditions (West et al., 2016; Reintjes et al., 2019a; Duerschlag et al., in revision).

The South Pacific Gyre (SPG), an oligotrophic gyre characterized by very low surface chlorophyll *a* concentration and extremely low inorganic nitrogen concentrations in the euphotic zone, covers nearly 10% of the surface ocean (Raimbault et al., 2008; Duerschlag et al., in revision). Due to its remote location, however, comparatively few studies have focused on heterotrophic carbon cycling in the oligotrophic SPG (Raimbault et al., 2008; Sempere et al., 2008; Letscher et al., 2015; Duerschlag et al., in revision). At three stations that were part of a larger transect through the SPG (Reintjes et al., 2019a; Duerschlag et al., in revision), we investigated microbially driven carbon cycling by measuring the initial step of heterotrophic organic matter degradation, extracellular enzymatic hydrolysis of high molecular weight (HMW) substrates. We focused on the hydrolysis of polysaccharides because they constitute a major fraction of phytoplankton biomass and thus of marine organic matter (Biersmith and Benner, 1998; Hedges et al., 2002).

Heterotrophic bacteria must initially use extracellular enzymes to hydrolyze HMW organic matter outside the cell to sizes suitable for further processing inside the cell. Enzymatic processing of polysaccharides can occur by two distinct mechanisms, via extracellular enzymes that are freely released or attached to the outer membrane, enzymes whose activities yield low molecular weight (LMW) hydrolysis products that are released into the external environment. These LMW hydrolysis products can potentially also be taken up by organisms that did not produce the enzymes; here, we term this mechanism of hydrolysis “external hydrolysis.” Another mechanism, initially discovered among members of the gut microbiome, is termed “selfish” uptake (Cuskin et al., 2015) because hydrolysis at the outer membrane is closely coupled to substrate uptake, such that large substrate fragments are transported intact into the periplasm, with little to no release of LMW hydrolysis products to the external environment. This mechanism has recently been shown to be widespread in surface waters of the ocean, also during spring phytoplankton blooms (Reintjes et al., 2017, 2019b, 2020). Here, we investigated the substrate specificities, rates of hydrolysis, and mechanisms by which polysaccharides are processed by heterotrophic bacterial communities in the oligotrophic SPG. Heterotrophic organisms play a key role in recycling of organic matter and thus also in regenerating the nutrients used to fuel primary productivity in the gyre. Few prior investigations have been made of enzyme activities in the central South Pacific (Koike and Nagata, 1997; Balmonte et al., 2020); to the best of our knowledge, there are no studies of extracellular enzyme activities in the SPG. In conjunction with our measurements of rates and mechanisms of polysaccharide processing, we tracked changes in bacterial community composition during the course of our incubations to reveal the links between bacterial community composition and function.

## Materials and Methods

### Study Sites and Sample Collection

**FIGURE 1:**
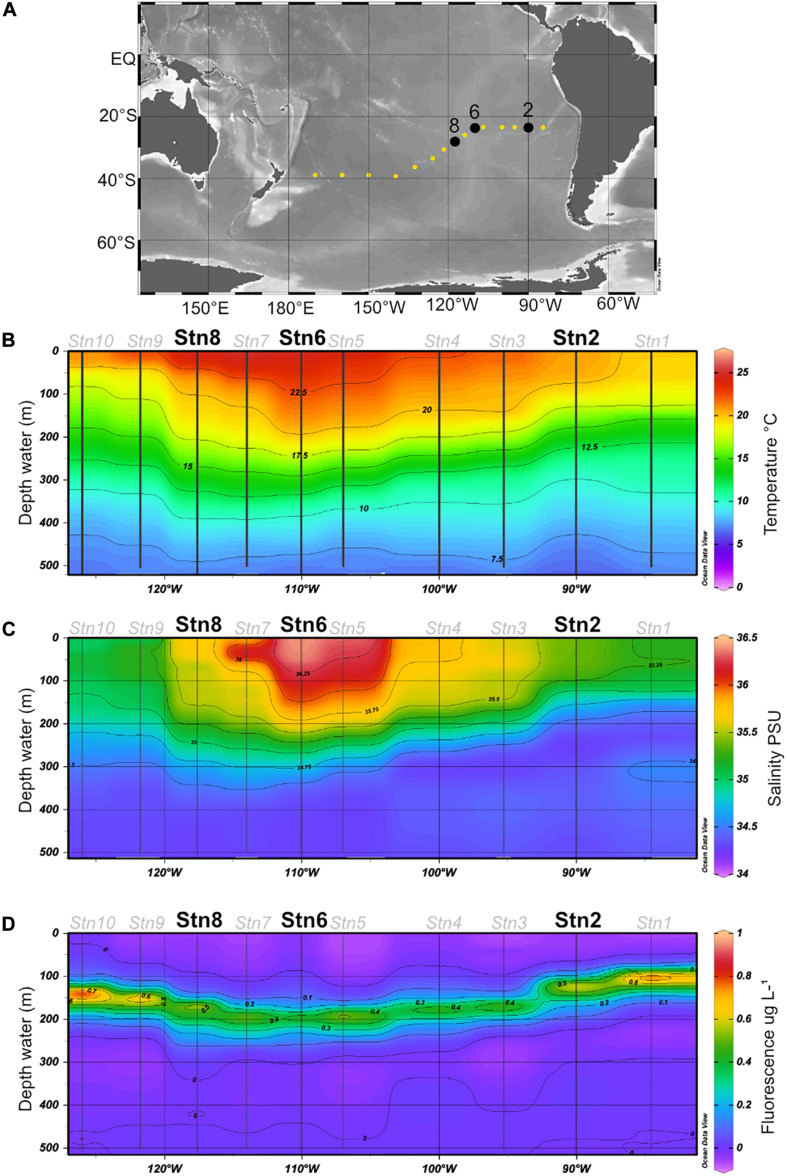
**(A)** Cruise track for the SO245 expedition; Stns 2, 6, and 8 are shown in black. **(B–D)** Temperature, salinity, and fluorescence in the upper 500 m of the water column, plotted from CTD data (see section “Materials and Methods”) obtained at Stns. 1–10. All figures were created using Ocean Data View software (Schlitzer, 2017).

Water samples were collected at station (Stn.) 2 (23.49°S, 90.03°W), Stn. 6 (23.50°S, 110.06°W) and Stn. 8 (27.27°S, 117.62°W) (Figure 1A) during R/V *Sonne* expedition SO-245 (December 2015–January 2016). These specific stations were chosen because they differ in their physicochemical conditions: Stn. 2 is an oligotrophic station of the SPG with irregular inputs of nutrients from coastal upwelling and low but measurable surface chlorophyll fluorescence, while Stns. 6 and 8 are ultraoligotrophic sites both in the center of the gyre, with higher temperatures and low fluorescence. Due to the length of incubations (see below) and the workload aboard ship, moreover, we could only carry out these incubations at a limited number of stations.

The water, as well as temperature, salinity, and chlorophyll fluorescence data (Figure 1B), were collected using a Seabird Sbe911 CTD attached to a Niskin rosette; for sample collection details see Reintjes et al. (2019a) and Zielinski et al. (2017). For these experiments, water was collected at 75 m at all three stations. This depth was above the deep chlorophyll maximum (DCM) at all three stations, but well within the euphotic zone (Duerschlag et al., in revision). At Stn. 8, additional samples were collected at depths of 160 m (in the euphotic zone, under the DCM), and also at depths of 1,250 and 2,800 m (aphotic zone). All water samples were collected from the Niskin bottles into sterile 1L acid washed glass bottles and directly used for substrate incubations.

### Incubations

Triplicate live substrate incubations plus one killed control (autoclaved seawater from the corresponding depth, to which fluorescently labeled polysaccharides (FLA-PS) were added after the water had cooled) were carried out. One of six FLA-PS (pullulan, laminarin, xylan, fucoidan, arabinogalactan, and chondroitin sulfate) was added to single incubations at a concentration of 3.5 μM monosaccharide-equivalent. These polysaccharides were selected because they are major components of marine algae and plankton, and/or enzymes hydrolyzing these polysaccharides have been demonstrated to occur in marine bacteria (e.g., Alderkamp et al., 2007; Neumann et al., 2015).

All samples were incubated in the dark at room temperature (ca. 20°C), including the samples from Stn. 8 which originated from 1,250 and 2,800 m. At select timepoints (0, 3, 6, 9, 12, 18 d), subsamples were collected from each incubation for microbial community analysis, for cellular abundance quantification, to quantify via fluorescence microscopy selfish uptake of polysaccharides, and to measure enzymatic hydrolysis rates. Samples for measurements of enzymatic activity were stored frozen (−20°C or −80°C) until processing, as described below.

For community analysis, 10 ml of water was gently extracted from each incubation using a sterile syringe and filtered through a 25 mm (0.2 μm pore size) polycarbonate filter (MilliporeSigma, Germany) using a Whatman 420200 Swin-Lok filter holder (MilliporeSigma). Two ml of the filtrate from these filtrations was collected for measurements of extracellular enzyme activities. The filters for microbial community analysis were stored at −20°C. For cellular abundance and selfish quantification, triplicate 20 ml of water samples were taken from the incubations and fixed for 1 h at room temperature (RT) with formaldehyde (1%). After fixation, the samples were filtered on a 47 mm (0.2 μm pore size) polycarbonate filter (MilliporeSigma) using a hand pump and a gentle vacuum of <200 mbar. All filters were air-dried at RT and then stored at −20°C.

### Measurement of Extracellular Enzyme Activities

FLA-PS were synthesized and characterized as described in detail in Arnosti (2003). In brief, polysaccharides purchased from Sigma (pullulan, laminarin, xylan, fucoidan, arabinogalactan, chondroitin sulfate) were activated with cyanogen bromide, injected on a Sephadex gel column (G-25 or G-50), and incubated overnight with fluoresceinamine. Labeled polysaccharides were separated from unreacted fluorophore using Vivaspin centrifugal concentrators. FLA-PS were characterized by measuring carbohydrate content (Chaplin and Kennedy, 1986) and by measuring absorbance at 490 nm against a standard curve. All of these FLA-PS are soluble.

Hydrolysis rates were measured as the change in polysaccharide molecular weight as a function of time, as determined via gel permeation chromatography with fluorescence detection (Arnosti, 2003). Unfortunately, a considerable number of samples for measurement of enzyme activities were lost in transit. Instead of complete time courses to measure enzyme activities, only a limited number of samples other than t0 were available for analysis: for Stn. 2 only the samples from 18 days; for Station 6, samples from 9, 12, and 18 days; and for Station 8, samples for 6, 9, 12, and 18 days. Note that the absence of the earlier timepoints means that hydrolysis rates calculations most likely significantly underestimate enzymatic activities. Since hydrolysis rates are calculated as the change in molecular weight distribution of the total added substrate with time (Arnosti, 2003), if hydrolysis is nearly complete at an early timepoint in the incubation, at later timepoints the calculated hydrolysis rates decrease, due to increasing elapsed time and a lack of changes in the molecular weight distribution of the FLA-PS. In any case, the calculated hydrolysis rates should be regarded as potential rates, since added substrate is in competition with any naturally occurring substrate for enzyme active sites.

### Cellular and Selfish Abundance Quantification

Bacterial total cell counts and selfish substrate uptake were quantified microscopically in every incubation and the treatment control at every timepoint by staining filtered cells (see above) with 4′,6-diamidino-2-phenylindole (DAPI, 1 ng/μl working solution, 7 min at RT in the dark). The filters were then mounted onto glass slides using a Citifluor/Vectashield (4:1) mounting solution. All samples were then visualized, and cell abundances enumerated using an automated image acquisition and enumeration system (Bennke et al., 2016). Briefly, the system consists of a Zeiss AxioImager.Z2 microscope stand (Carl Zeiss GmbH, Germany) with a cooled charged-coupled-device (CCD) camera (AxioCam MRm, Carl Zeiss) and a Colibri LED light source (Carl Zeiss) with three light-emitting diodes (UV-LED: 365 ± 4.5 nm for DAPI, blue-LED: 470 ± 14 nm for FLA-PS (488), red-LED: 590 ± 17.5 nm for autofluorescence). Images are obtained from a defined set of coordinates consisting of a minimum of 36 fields of view on each filter. They are acquired using a 63× oil emersion plan apochromatic objective (Carl Zeiss), at selected wavelengths and with fixed exposure times. Afterward the images are analyzed in the ACMEtool2^1^ image analysis software. From the DAPI images, cells are defined by fixed parameters (such as signal to background ratio and area) and subsequently enumerated. Positive substrate stained cells (“selfish”) are defined by a positive signal in both the DAPI and FLA-PS (488) images, with a minimum overlap of 30% and a fixed area size between 17–30 pixel (0.17 – 0.3 μm^2^) and a minimum signal to background ratio of 1 to 2.5. All automated image results were also manually curated using the same images; this is important to access potential false positive FLA-PS signals.

### Microbial Diversity Analysis

All substrate incubations and the treatment controls were 16S rRNA gene sequenced. Total DNA was extracted from each sample filter using the DNeasy Power Water Kit (Qiagen) according to the manufacturer’s recommendations. PCR was carried out using the primers S-D-Bact-0341-b-S-17 (5′-CCT ACG GGN GGC WGC AG-3′) and S-D- Bact-0785-a-A-21 (5′-GAC TAC HVG GGT ATC TAA TCC-3′) targeting the V3-V4 variable region of the 16S rRNA gene (Klindworth et al., 2013). The forward primer was barcoded with one of 40 IonXpress barcodes (IonXpress 1 to 40, Thermo Fisher) to enable the multiplexing of samples. After PCR the amplicons were size-selected using the Agencourt AMPure XP magnetic beads (Beckman Coulter, Germany) and quantified on a fragment analyzer (AATI) using the DNF-472 standard sensitively NGS sizing kit (AATI). The amplicons were then pooled equimolarly into sequencing libraries and prepared for sequencing using the Ion PGM Hi-Q OT2 kit (Thermo Fischer) on a OneTouch 2 (Thermo Fisher) and One Touch ES instrument (Thermo Fisher). The produced library fragments were sequenced using the Ion PGM Hi-Q sequencing kit (Thermo Fisher) on an Ion Torrent PGM platform (Thermo Fisher). Sequencing was done with Ion 316 v2 chips (6.1 million sensors and 1 Gb output, Thermo Fisher) with a total of 1,200 flows per sequencing run.

The Torrent Suite software (Thermo Fisher), which converts the raw signals (raw pH-values) into base calls for each read, was used for quality trimming (settings: BaseCaller – barcode-mode 1 – barcode- cutoff 0 –trim-qual-cutoff 15 –trim-qual-window-size 10 –trim-min-read-len 250). The data was exported as SFF files, split into individual FASTA files using mothur version 1.35.1 (Schloss et al., 2009) and classified against the SILVA SSU Ref dataset (release 132) using the SILVAngs platform (Quast et al., 2013).

A total of 116 samples were analyzed, yielding nearly 8 million reads with an average length of 406 bp. The average read abundance per samples was 65,000 with a minimum of 10,000 and a max of 280,000 reads. Nine samples, although repeatedly sequenced, had a read abundance under 10,000. These included several of the t0 deep incubation samples which had a low cellular abundance and correspondingly low DNA yield. The samples were still included in the analysis but could show a reduced diversity and therefore affect the community analysis.

The change in community composition over the course of each incubation was analyzed and visualized using R version 3.6.3 (2020-02-29; R Core Team, 2013) in Rstudio version 1.2.5033 (R Studio Team,, 2019) with the packages Vegan, Corrplot, and Rioja (Juggins, 2017; Wei and Simko, 2017; Oksanen et al., 2019). The read abundances were normalized by dividing by the sample total. Beta-diversity hypothesis testing was done using Bray-Curtis dissimilarity matrices of the total bacterial community of each sample. Tests for significant difference in the bacterial community composition across sampling time points within a substrate and between substrates, as well as across stations and depth, were performed by analysis of similarity (ANOSIM) and permutation multivariant analysis of variance (PERMANOVA).

## Results

### Environmental Description

Water samples were collected at Stn. 2, at the easternmost edge of the SPG, and at Stns 6 and 8, within the center of the gyre (Figure 1). Both the *in situ* temperature and salinity at a depth of 75 m differed somewhat for Stns 2, 6, and 8 (Figure 1), with the highest salinity and *in situ* temperature at Stn. 6. At all three stations, very little chlorophyll was measurable above a depth of 70 m; the 75 m depth horizon was close to the depth at which chlorophyll concentrations just began to be measurable (Reintjes et al., 2019a). The 1% light layer (usually defined as the extent of the euphotic zone) typically extended down to 162 m in the gyre; and the DCM (deep chlorophyll maximum) was located somewhat below this depth at Stns. 2, 6, and 8 (Reintjes et al., 2019a).

### Enzymatic Activities

**FIGURE 2:**
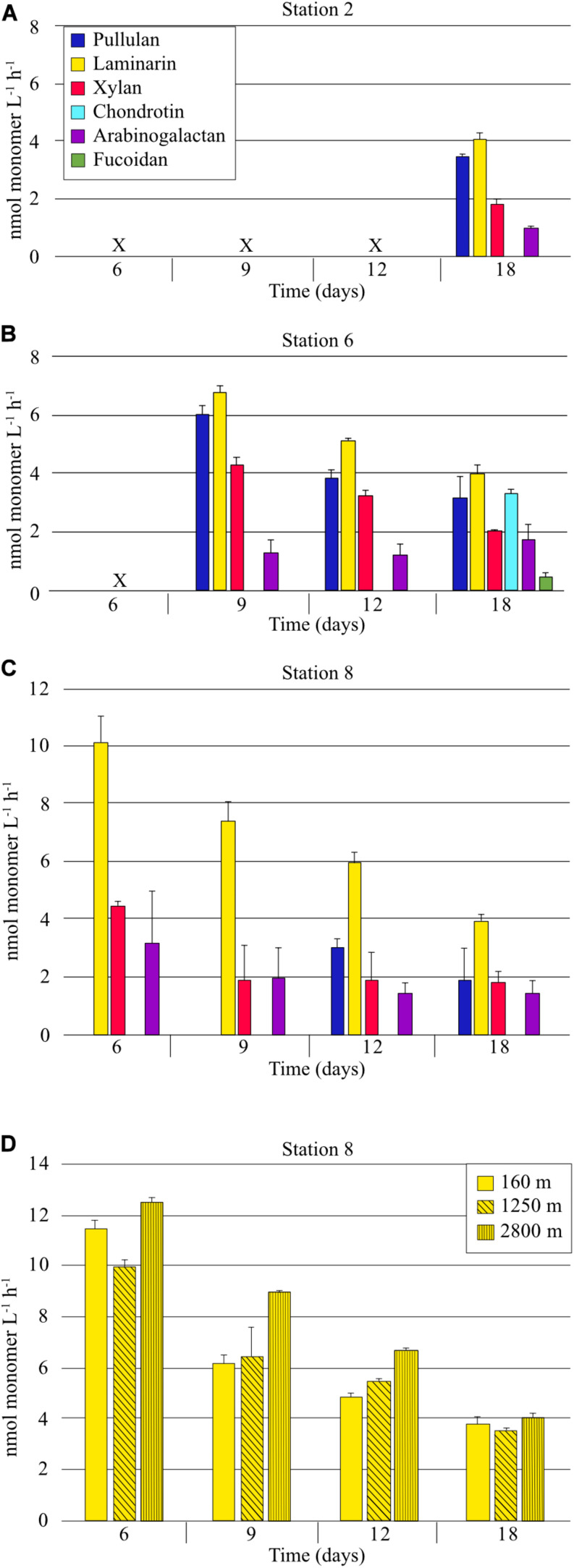
Extracellular enzymatic hydrolysis rates of polysaccharides in samples collected from a depth of 75 m at **(A)** Stn. 2 **(B)** Stn. 6, **(C)** Stn. 8. **(D)** Extracellular hydrolysis rates of laminarin in water collected from depths of 160, 1250, and 2800 m at Stn. 8. X indicates that no sample was available (see section “Materials and Methods”). Error bars show standard deviation between triplicate incubations.

Six FLA-PS were used to measure polysaccharide processing capabilities of heterotrophic bacterial communities. At Stn. 2, on day 18, the only incubation day for which enzyme activity samples were available (see section “Materials and Methods”), four of the six polysaccharides were hydrolyzed; calculated hydrolysis rates of pullulan, laminarin, xylan, and arabinogalactan averaged 3.5 nmol monomer L^–1^ h^–1^, 4.1 nmol monomer L^–1^ h^–1^, 1.8 nmol monomer L^–1^ h^–1^, and 1.0 nmol monomer L^–1^ h^–1^, respectively (Figure 2). These rates are likely an underestimate, due to the late timepoint for which we have samples. At Stn. 6, in contrast to Stns. 2 and 8, all six polysaccharides were hydrolyzed; calculated hydrolysis rates of pullulan, laminarin, and xylan decreased with time from days 9 to 18 due to the fact that hydrolysis was nearly complete by day 9, and thus are likely also underestimated. Arabinogalactan hydrolysis rates were generally constant over this time period, and fucoidan and chondroitin hydrolysis were first detectable at day 18. At Stn. 8, the same four polysaccharides – pullulan, laminarin, xylan, and arabinogalactan – were hydrolyzed as at Stn. 2. Hydrolysis rates of these polysaccharides generally also decreased with time, because hydrolysis was nearly complete by the first timepoint for which we have samples (day 6). At day 18, the only date for which we have data from all three stations, hydrolysis rates were comparable for pullulan, laminarin, xylan, and arabinogalactan. Fucoidan and chondroitin were measurably hydrolyzed only at Stn. 6 (Figure 2).

Laminarin hydrolysis was additionally measured from samples collected at depths of 160, 1,250, and 2800 m at Stn 8. All samples were incubated at the same temperature (ca. 20°C). For these incubations, samples were available from the 6 day timepoint onward, when the communities had shifted very substantially from their initial cell numbers and composition (see below). At the 6 day timepoint, laminarin hydrolysis was nearly complete (hydrolysis rates of 11.5, 10.0, and 12.6 nmol monomer L^–1^ h^–1^ for 160, 1,250, and 2,800 m, respectively); laminarin hydrolysis rates at the 18 day timepoint were comparable to those measured at the other stations (Figure 2).

### Microbial Cell Counts

**FIGURE 3:**
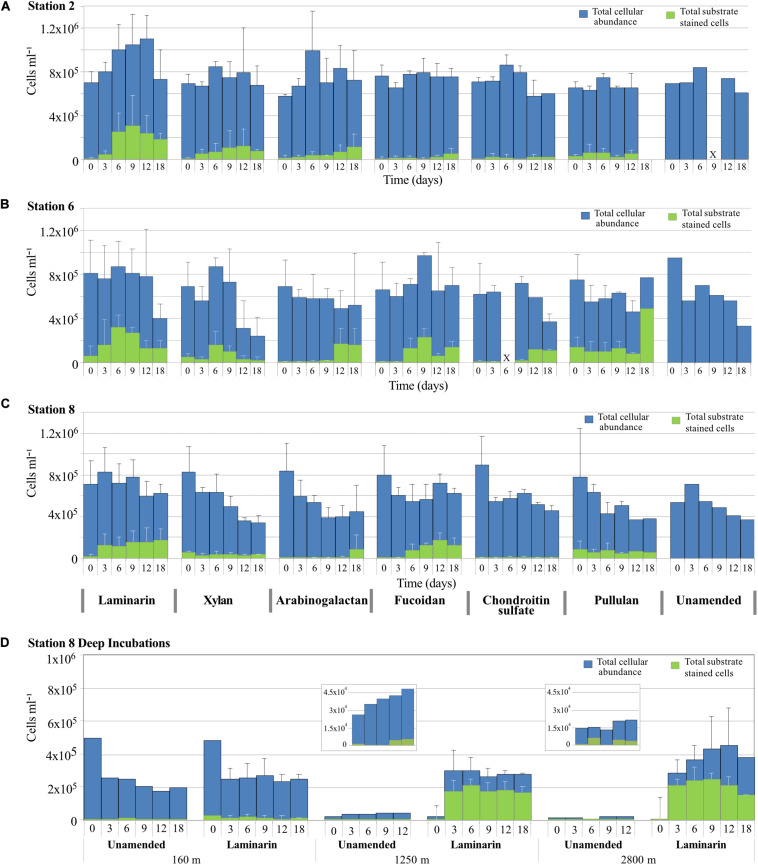
Total cell counts (DAPI staining; shown in blue) and the number of cells showing selfish staining (staining with FLA-PS; shown in green) over time for incubations with different polysaccharides, plus the no-amendment incubation at **(A)** Stn. 2, **(B)** Stn. 6, **(C)** Stn. 8, and **(D)** depths of 160, 1,250, and 2,800 m at Stn. 8. Error bars show standard deviation between total cell counts from triplicate incubations.

Cell counts from all incubation with water from 75 m initially were generally between 6 × 10^5^ cells ml^–1^ and 8 × 10^5^ cells ml^–1^ (Figure 3). At Stn. 2, aside from an increase in average cell counts during the first 12 days of the laminarin incubation, total cell counts remained relatively constant during the time course of the incubation. At Stn. 6, there was a pronounced decline in cell counts at later timepoints (at d18, or starting at d12) for the laminarin, xylan, chondroitin, and unamended incubations, with final cell counts close to 3 × 10^5^ cells ml^–1^. Cell counts in the pullulan, arabinogalactan and fucoidan incubations varied somewhat, but did not show a comparably sharp decrease. At Stn 8, cell counts also declined starting at the 6 or 9 days timepoint, especially for the xylan, arabinogalactan, pullulan, and unamended incubations. Patterns of cell counts in unamended incubations were similar to the amended incubations; incubations that showed little to no extracellular enzymatic activity (e.g., fucoidan and chondroitin at Stns. 2 and 8) showed the same cell count patterns as the other incubations.

The cell counts in the Stn. 8 depths samples showed very distinct patterns: the 160 m incubation showed a very sharp decrease in cell numbers in both the laminarin and the unamended incubations, from initial cell counts close to 5 × 10^5^ cells ml^–1^ down to 2.5 × 10^5^ cells ml^–1^ starting at the 3 day timepoint, suggesting extensive cell losses due to viruses or grazing. The deep incubations at Stn. 8, with water from 1,250 and 2,800 m, showed a completely different pattern: initially low cell numbers in the laminarin-amended incubations increased by well over an order of magnitude, from initial counts of 2 × 10^4^ cells ml^–1^ at 1,250 m and 1.5 × 10^4^ cells ml^–1^ at 2,800 m to close to 3 × 10^5^ cells ml^–1^ at day 3 for water from both depths. Cell counts in unamended incubations from 1,250 m also increased (from 2.6 × 10^4^ cells ml^–1^ to 3.5 × 10^4^ cells ml^–1^ at day 3), whereas cell numbers in the unamended incubation from 2,800 m remained constant at 1.5 × 10^4^ cells ml^–1^. Incubation at warmer temperature (20°C, compared to ca. 2–3°C *in situ*) combined with substrate addition therefore greatly increased overall cellular abundance only for water obtained from depths of 1,250 and 2,800 m; the warmer temperature was evidently sufficient, however, to also help increase cell numbers at 1250 m (Figure 3).

### Selfish Substrate Uptake

Bacteria that transport large polysaccharide fragments into their periplasmic space can be visualized and counted via fluorescence microscopy. Selfish uptake was considerable at all three stations, but the extent and timing of selfish uptake differed by substrate and station (Figure 3). Selfish uptake at the t0 timepoint (equivalent to ca 15 min of exposure to substrate) likely reflects the *in situ* microbial community, whereas selfish uptake at later timepoints in the incubation can be a function of changed community composition and activity with extended incubation time.

At Stn. 2 at the t0 timepoint, very few cells initially stained. However, the number of substrate stained cells increased markedly with incubation time especially for laminarin, but also for xylan and arabinogalactan, with low numbers of cells stained with fucoidan and chondroitin. At Stn. 6, in contrast, a much higher number of cells were stained at the t0 timepoint with laminarin, xylan, and pullulan, and throughout the time course of the incubations, a larger number of cells showed selfish uptake, especially of fucoidan, chondroitin, arabinogalactan, and pullulan. All six substrates thus were taken up selfishly by a considerable number of bacteria at Stn. 6. At Stn. 8, selfish uptake at the t0 timepoint was measurable for the xylan, pullulan, and laminarin incubations. Over the time course of the incubations, the number of cells showing selfish substrate uptake increased considerably in the laminarin and fucoidan incubations, and remained relatively constant in the xylan and pullulan incubations. At the 18d timepoint, a considerable number of cells also showed selfish uptake of arabinogalactan.

The depth profile from the laminarin incubations at Stn. 8 showed striking differences: a small fraction of the cells at 160 m took up laminarin from the t0 timepoint onward. At depths of 1,250 and 2,800 m, in contrast, starting after 3 days’ incubation, approximately 2 × 10^5^ cells ml^–1^ (on average 61% of cells) showed selfish uptake of laminarin, a high fraction of the total cells that remained substrate stained throughout the incubation.

### Bacterial Community Analysis

**FIGURE 4:**
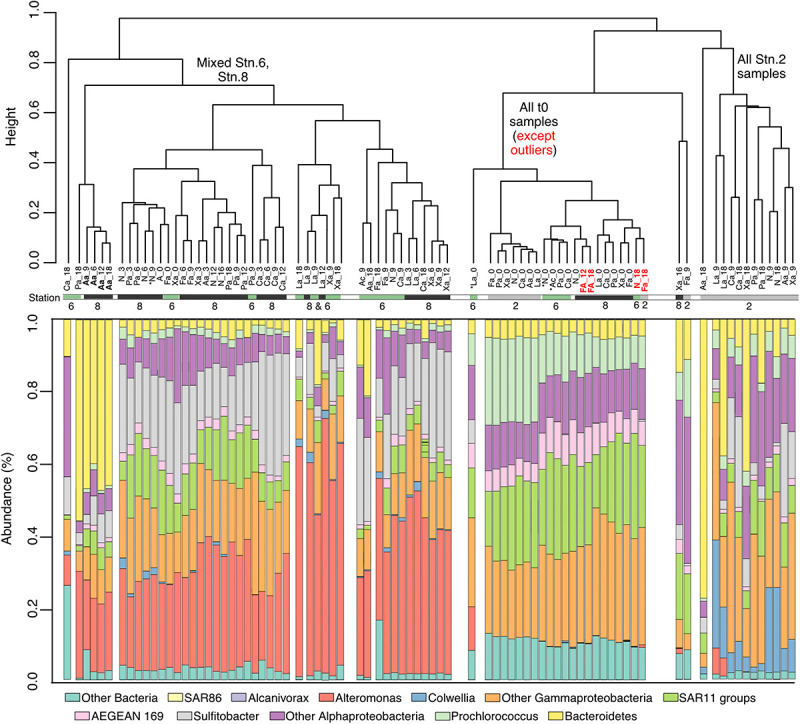
Dendrogram showing the similarity clustering of microbial community compositions between samples from different stations, incubations, and time points. Bar charts underneath the dendrogram shows relative community composition of each incubation and time point. ^∗^ indicates samples with <10,000 reads. Abbreviations under dendrogram branches indicate substrate and incubation timepoint (e.g., La_9 indicates laminarin incubation at 9 days).

The bacterial community composition between Stns. 2, 6, and 8 was initially very similar – aside from a few outliers – but changed with time in amended as well as unamended incubations (Figure 4 and Supplementary Figure 1). The t0 communities were characterized by the presence of SAR11, AEGEAN-169, *Prochlorococcus* and a range of *Alphaproteobacteria* and *Gammaproteobacteria*. During the incubations all of the incubations from Stn. 2 separated from the Stn. 6 and Stn. 8 incubations (Figure 4 and Supplementary Figure 2). The communities from Stns. 6 and 8 were characterized by a very large relative contribution of *Alteromonas*, which was not among the abundant sequences at the initial time point, but with time became a very prominent fraction of the sequences (Figure 4). *Alteromonas* also became abundant in the unamended controls of Stns. 6 and Stn. 8. In addition, *Sulfitobacter* increased in relative abundance, and a range of *Gammaproteobacteria*, and *Alphaproteobacteria* continued to be high in abundance at the later timepoints (Supplementary Figure 1). The microbial community of Stn. 2 separated considerably from that of Stns. 6 and 8, with communities differing significantly by timepoint, as well as station (Supplementary Figure 2).

Substrate-specific changes in community composition were also evident for some of the amended incubations. The arabinogalactan incubations from Stn. 8 starting at the 6 day timepoint contained a very high relative proportion of *Bacteroidetes* sequences (*Leeuwenhoekiella*), and were thus dominated by *Alteromonas* and *Bacteroidetes*. At Stn. 2, *Gramella* became comparatively more abundant in the arabinogalactan and also (to a lesser extent) in the laminarin incubation. Furthermore, other *Bacteroidetes* became comparatively more abundant in the pullulan incubation (*Croceibacter and Winogradskyella*), and in the xylan incubation (*Salegentibacter*) (Supplementary Figure 1). Although the incubations for Stn. 6 showed enrichment in *Alteromonas* for most substrates, *Leeuwenhoekiella* was relatively more enriched in the pullulan and arabinogalactan incubation.

**FIGURE 5:**
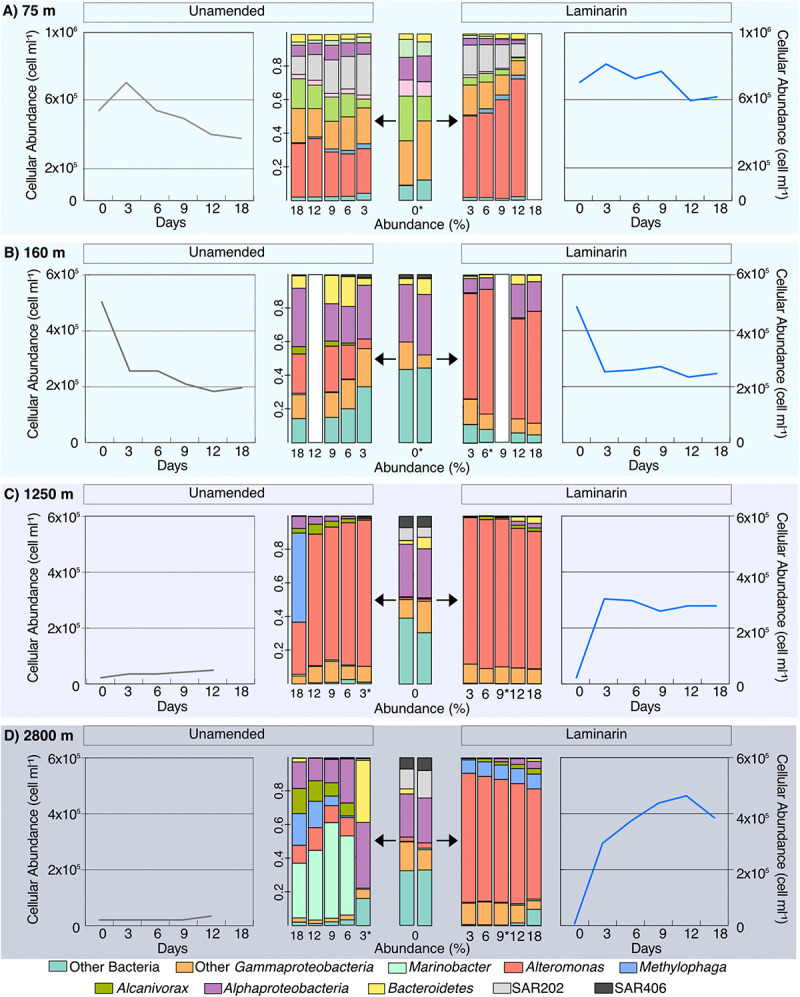
Community composition of laminarin incubations and unamended incubations from depths of **(A)** 75 m; **(B)** 160 m; **(C)** 1,250 m; and **(D)** 2,800 m from Stn. 8. Central panels show changes in community composition with time, evolving for laminarin-amended incubations to the right, and for the unamended incubations to the left. Changes of cell counts in the same incubations, with time, flank the community composition. White bars indicate no community composition data is available and ^∗^ indicate samples with <10,000 reads.

At Stn. 8, initial bacterial community composition from the water collected at 160 m included sequences from *Bacteroidetes*, *Alphaproteobacteria*, and *Gammaproteobacteria*; the samples from 1,250 and 2,800 m additionally showed larger relative contributions of SAR406, plus SAR202, and a small contribution of *Alteromonas* (Figure 5, Supplementary Figures 3, 4). The laminarin-amended incubations from all depths, starting at the 3d samples, were dominated by *Alteromonas*, a change that was also seen, however, for the unamended samples from 1250 m (these were the samples that also showed considerable relative increase in bacterial numbers; Figure 5). By contrast, the unamended incubations from 160 m stayed relatively close to the initial composition (although a contribution of *Alteromonas* became more evident), and the unamended incubations from 2500 m showed a substantial contribution of *Marinobacter* (Figure 5, Supplementary Figure 4).

## Discussion

The SPG covers a major portion of the Earth’s surface. Still, in part due to its remote location, only a handful of investigations have focused on biogeochemical processes and their microbial drivers within the SPG. These studies have determined that the photosynthetic community consists particularly of Prochlorococcus, aerobic anoxygenic phototrophs, and a diverse community of small photosynthetic eukaryotes (Grob et al., 2007; Lami et al., 2007; Duerschlag et al., in revision). Photosynthetic activity occurs throughout the euphotic zone, rather than being centered at the DCM, with 68–79% of primary productivity taking place above the DCM (Duerschlag et al., in revision). Similarly, bacterial productivity is evenly spread throughout the euphotic zone, and is comparable with other subtropical and temperate environments (Van Wambeke et al., 2008). Integrated primary productivity of the euphotic zone amounts to 20 mg C m^2^ d^–1^, somewhat higher than previous estimates derived only from satellite-measured surface chlorophyll (Duerschlag et al., in revision). Within the euphotic zone, particulate organic carbon, particulate organic nitrogen and particulate organic phosphorus do not show any distinct depth trends, and thus are not correlated with chlorophyll a concentrations (Raimbault et al., 2008). The extremely low inorganic nitrogen concentrations throughout the euphotic zone (Raimbault et al., 2008), however, also reflect the importance of recycled nutrients for primary production in the SPG, and thus the importance of the heterotrophic activities that are responsible for recycling organic matter.

### Station-Related Differences in Polysaccharide Processing

The considerable enzyme activities measured at Stns. 2, 6, and 8 (Figure 2) of the SPG are consistent with heterotrophic communities capable of actively recycling organic matter, and are comparable to those measured in a wide range of other open-ocean settings (Arnosti et al., 2011; Reintjes et al., 2019b). However, they also demonstrate station-related differences in functional capabilities of the heterotrophic communities. In particular, all six polysaccharide hydrolase activities were measurable at Stn. 6, whereas only pullulan, laminarin, xylan, and arabinogalactan were externally hydrolyzed at Stns. 2 and 8. The broad spectrum of enzyme activities at Stn. 6 is remarkable, since this pattern has only been observed in a few other locations in the ocean, including two stations in the west-central Pacific (168°W, 7°S; 161°W, 15°N), a few stations in the Gulf of Mexico (Arnosti et al., 2005, 2009; Steen et al., 2012), and several stations in the nearshore northwestern Atlantic (D’Ambrosio et al., 2014; Bullock et al., 2015), locations that are quite different in physical-chemical characteristics from the SPG. By comparison, two stations in more productive coastal waters off South America outside the SPG (75.0°W, 26.3°S; 79.2°W, 23.1°S) showed hydrolysis profiles similar to those seen at Stns. 2 and 8, with the same four substrates hydrolyzed at similar rates (lam > xyl > ara, pull) (Arnosti et al., 2011). The other remarkable observations with respect to enzyme activities relates to chondroitin and fucoidan: chondroitin hydrolysis is comparatively common in the upper water column in the ocean, but fucoidan hydrolysis is relatively rare (Arnosti et al., 2011). Thus, the measurable fucoidan hydrolysis at Stn. 6 is notable, as is the lack of chondroitin hydrolysis at Stns. 2 and 8.

The remarkable breath of polysaccharide utilization and station-related patterns also extended to selfish substrate uptake (Figure 3). Selfish uptake was most significant in absolute as well as relative cell numbers at Stn. 6, where all six polysaccharides were taken up. At Stn. 2, in contrast, only laminarin and xylan, and – at later incubation time points – arabinogalactan were taken up by a high number of cells. Similarly, at Stn. 8, selfish uptake was more selective, and the more-targeted substrates were laminarin, fucoidan, and pullulan, with late (day 18) selfish uptake of arabinogalactan. Overall, microbially driven polysaccharide processing was distinct at all three stations. Both Stns. 2 and 8 had lower selfish activity and a narrower range of externally hydrolyzed substrates. Stn. 8 additionally showed higher fucoidan selfish uptake, which intriguingly showed no corresponding external hydrolysis; Stn. 6 had the highest and broadest range of polysaccharide processing capabilities.

These capabilities at Stn. 6 – including comparatively high initial (t0) selfish substrate uptake – may be due to the presence of diatoms. At a depth of 62 m at Stn. 6, diatoms were found to contribute 5% of the relative OTU abundance of the eukaryotic community. Their contributions at the same depth at Stns. 2 and 8 were 0.25 and 1%, respectively (Duerschlag et al., in revision). Plankton cell counts from vertical net tows during the SO-245 cruise, integrating the upper 300 m of the water column, showed that diatoms constitute 10, 3.5, and 13.5% of large cells (>20 μm) at Stns. 2, 6, and 8, respectively (Heinzmann, 2016). The lower integrated cell numbers but higher OTU abundance at Stn. 6 (Duerschlag et al., in revision) suggests that the diatom population was likely concentrated close to the 75 m depth horizon, the same water depth used for FLA-PS incubations. Ras et al. (2008) similarly noted an increase in relative concentration of fucoxanthin (a diagnostic pigment of diatoms) in the upper 80 m of the water column in the center of the SPG, suggesting that their presence at this depth may be a recurrent feature of the phytoplankton community.

The presence of diatoms may be linked to polysaccharide processing because diatoms excrete carbohydrate-rich DOM (Biersmith and Benner, 1998); a higher relative abundance of diatoms may lead to greater exposure to complex polysaccharides. These polysaccharides include not only laminarin (an energy storage polysaccharide of diatoms; Gügi et al., 2015), but also fucose-containing sulfated polysaccharides (Vidal-Melgosa et al., in revision). Since pre-exposure to a substrate has been shown to increase rates of initial selfish substrate uptake (Reintjes et al., 2017), the presence of diatoms could explain considerable selfish uptake and the generally high polysaccharide processing capabilities at Stn. 6. Spatial differences in selfish uptake and external hydrolysis at Stns. 2, 6, and 8 might therefore be linked to the presence and abundance of specific phytoplankton-derived substrates, an observation consistent with temporal changes in patterns of selfish uptake and external hydrolysis over the course of a phytoplankton bloom in the North Sea (Reintjes et al., 2020).

### Changes in Bacterial Communities

The composition of heterotrophic microbial communities differed substantially by station and incubation timepoint, with communities that initially shared a considerable degree of similarity developing compositional differences that were amplified through the course of the incubations (Figure 4; Supplementary Figure 2). At t0, communities from Stns. 6 and 8 clustered together in a dendrogram, with Stn. 2 forming a distinct but related cluster (Figure 4); for all incubations – amended and unamended – the Stn. 2 cluster developed distinctly from Stns. 6 and 8. In particular, *Alteromonas* and *Sulfitobacter*, rare among the initial communities, became more abundant during the incubation from Stns. 6 and 8 (Figure 4, Supplementary Figure 1), whereas *Sulfitobacter* and *Collwellia* developed in Stn. 2 samples. This pattern – a change in community composition driven by initially minor community members - is consistent with a previous investigation of bacterial community composition across the SPG, which found considerable regional differences among western, central and eastern groups, especially among the less-abundant taxa (Walsh et al., 2015).

The development of *Alteromonas* in incubations from water collected at 75 m at Stns. 6 and 8 was also seen in the incubations with samples from 160, 1250, and 2800 m at Stn. 8 (Figure 5). Water from these depths likely originate from distinctly different water masses, as demonstrated by their different physical and chemical characteristics. The 1250 m sample was likely a mixture mostly of Pacific Deepwater with Antarctic Intermediate Water (Fitzsimmons et al., 2016), and the 2800 m water a mix of Pacific Deepwater with Circumpolar Deep Water (Peters et al., 2018). From these waters of different origin, showing somewhat different initial community compositions, the initially low abundance of *Alteromonas* changed rapidly, constituting ca. 50–80% of the total sequence abundance in amended incubations (and in the unamended incubations from 1250 m; Figure 5; Supplementary Figures 3, 4), starting at the d3 timepoint. These *Alteromonas* likely are ‘opportunitrophs’ (Polz et al., 2006), “boom-and-bust” specialists that respond rapidly to changes in conditions associated with bottle incubations.

### Mechanisms of Laminarin Processing: Especially Versatile *Alteromonas*?

The incubations with water from Stn. 8 also provide an unexpected view into mechanisms of laminarin processing. The comparatively high (at 6 days, 10–12 nmol monomer L^–1^ h^–1^; Figure 2) external hydrolysis rates in water from all depths is likely due to the fact that incubations were carried out at the same temperature (ca. 20°C); laminarin hydrolysis correlates tightly with environmental (in this case incubation) temperature (Arnosti et al., 2011). The rate of laminarin hydrolysis *in situ* can be approximated by considering the effects of temperature as well as rapid community growth. The temperature-laminarin hydrolysis rate equation (*y* = 0.5644x + 0.5098; *r*^2^ = 0.7695) derived from data of Arnosti et al. (2011) corrects the potential hydrolysis rates from 12 and 10 nmol monomer L^–1^ h^–1^ to 2.3 and 1.5 nmol L^–1^ h^–1^ at 1,250 and 2,800 m, respectively, based on the *in situ* temperatures of 3.19 and 1.5°C. Massive growth of *Alteromonas* also clearly contributed to laminarin hydrolysis; if we correct for the increase in total cell counts after 3 days’ incubation with laminarin (initial cell numbers at 1,250 and 2,800 m were 9.1% and 5%, respectively, of cell numbers at 3 days), we reach an approximation of 212 and 75 pmol monomer L^–1^ h^–1^ for the *in situ* external hydrolysis rate of laminarin at 1,250 and 2,800 m, respectively. In any case, incubating the water from 1,250 and 2,800 m at a temperature much in excess of *in situ* effectively gave us an enrichment of organisms able to cope with the abrupt change. Some of the organisms contained in the original water sample (Figure 5) – particularly the *Alteromonas* – clearly thrived under these changed conditions. These *Alteromonas*-dominated communities from 1,250 and 2,800 m also showed intensive selfish uptake, with 30–69% of total DAPI-stainable cells selfishly taking up laminarin (Figure 3). These lines of evidence – strongly *Alteromonas*-dominated communities, with considerable external hydrolysis as well as extensive selfish substrate uptake – imply that these organisms are capable of both mechanisms of polysaccharide processing. This is the first indication in a marine sample of both external hydrolysis and selfish uptake being used by the same organism, although gut-associated selfish *Bacteroidetes* that also carry out external hydrolysis have been identified (Rakoff-Nahoum et al., 2016). Note that we attribute the much lower fraction of selfish bacteria in the sample from 160 m to the observation that cell numbers in those incubations decreased by ca. 50% between the initial timepoint and the first sample point at 3 days, a drop observed also in the unamended incubation (Figure 3). The 160 m sample is very close to the DCM, where autotrophic picoeukaryotes – organisms that are frequently mixotrophic – were comparatively abundant (Duerschlag et al., in revision). Along with viruses, they may be responsible for the decrease in cell numbers in the 160 m incubations. Rapid cell growth and lysis may thus have prevented accumulation of laminarin in the periplasmic space of bacteria, and thus the identification of selfish bacteria.

Whether in marine systems a combination of selfish uptake and external hydrolysis in a single organism is typical for processing of laminarin remains to be determined. Laminarin is a remarkably abundant polysaccharide in the ocean (Alderkamp et al., 2007; Becker et al., 2020), which appears to be hydrolyzed virtually everywhere in the ocean (Arnosti et al., 2011; Balmonte et al., 2020). High rates of hydrolysis concurrent with extensive selfish uptake of laminarin have been measured in the late phases of a spring algal bloom in the North Sea; laminarin was the only polysaccharide for which such dynamics were observed (Reintjes et al., 2020). In the North Sea, however, *Colwellia* rather than *Alteromonas* was a dominant gammaproteobacterial responder, and *Gammaproteobacteria* were not particularly abundant among the selfish bacteria identified (Reintjes et al., 2020). Since the enzymatic capabilities of closely related bacteria can differ considerably (Xing et al., 2014; Avci et al., 2020; Koch et al., 2020) further investigation of the *Alteromonas* from the SPG would confirm whether they are indeed capable of both selfish uptake and external hydrolysis.

Simultaneous rapid selfish uptake and external hydrolysis may be specific to bacterial processing of laminarin not only because of its abundance, but also because it is structurally less complex than many other marine polysaccharides (Sichert et al., 2020). Moreover, enzymes to hydrolyze laminarin – and the capability to use laminarin as a substrate – are widely distributed among marine bacteria (Alderkamp et al., 2007; Kruger et al., 2019). Other – particularly more structurally complex – polysaccharides might not be externally hydrolyzed and selfishly taken up by the same organism. Fucoidan, for example, was selfishly taken up at Stns. 2 and 8, but not externally hydrolyzed. Similar observations have been made for fucoidan in the Atlantic Ocean (Reintjes et al., 2019b), and arabinogalactan uptake without measurable external hydrolysis has also been reported for the North Sea (Reintjes et al., 2020).

## Summary

Close coupling between autotrophic organic matter production and heterotrophic degradation is likely a central feature of the SPG, yielding nutrients required for continued primary production. Since complex biopolymers are integral constituents of living biomass, the broad capabilities of heterotrophic communities to recycle these biopolymers should perhaps not be so surprising. This region evidently includes not only organisms capable of existence on the fine edge of minimal substrate concentrations, but also those well-suited to effectively process high molecular weight organic matter, and capable of taking advantage of abrupt changes in physical conditions and substrate availability. Recent evidence for the input of polysaccharides to the high molecular weight DOM inventory of the deep ocean (Broek et al., 2020) provides a clue as to how such organisms may survive under the conditions that they experience *in situ*. Throughout the water column, distinct mechanisms of polysaccharide processing – selfish uptake and external hydrolysis – are evidently effective for substrates of widely differing structures, under a broad range of environmental conditions and substrate concentrations (Arnosti et al., 2021). Closer study of the organisms responsible for this activity, coupled with structural characterization of their target substrates, will help fill in the pieces of this emerging picture.

## Data Availability Statement

All sequence data were deposited in the European Nucleotide Archive (ENA) (Toribio et al., 2017) using the data brokerage service of the German Federation for Biological Data (GFBio) (Diepenbroek et al., 2014), in compliance with the MIxS standard (Yilmaz et al., 2011). The INSDC accession number for the data is PRJEB40503. All cell count data are publicly available from Pangaea (51): https://doi.pangaea.de/10.1594/PANGAEA.922868; enzyme activity data are also being deposited at Pangaea under the same doi.

## Author Contributions

GR, BF, RA, and CA planned experiments and data analysis. GR carried out shipboard sampling and experiments and post-cruise NGS sequencing and microscopy. CA synthesized the FLA-PS and analyzed the enzyme activity samples. GR and CA wrote the manuscript, with input from BF and RA. All authors contributed to the article and approved the submitted version.

## Conflict of Interest

The authors declare that the research was conducted in the absence of any commercial or financial relationships that could be construed as a potential conflict of interest.

## Footnotes

1 http://www.technobiology.ch/index.php?id=acmetool

## Supplementary Material

Supplementary Figure 1 Bubble plot of bacterial genera with a minimum normalized read abundance of 0.5%, in FLA-PS incubations and no-substrate incubation from Stns. 8, 6, and 2. Major phylogenetic groupings are shown on the left; bubble size corresponds to relative abundance in an individual FLA-PS incubation at each timepoint. L = laminarin, X = xylan, C = chondroitin sulfate, A = arabinogalactan, F = fucoidan, P = pullulan, N = unamended incubation. Small numbers at the bottom of the panel indicate incubation time in days (0 to 18 days).

Supplementary Figure 2 NMDS ordination showing Bray-Curtis dissimilarity in bacterial community composition during time course incubations from Stns. 2, 6, and 8. Ovals indicate standard deviation ellipse with a confidence limit of 0.5. Initial community composition (marked t0 and shown in smaller symbols) groups together; with time, the Stn. 2 incubations become more distinct from the Stn. 6 and 8 incubations.

Supplementary Figure 3 Bubble plot of bacterial genera with a minimum normalized read abundance of 0.5% for all incubations and timepoints from Stn. 8 with water from depths of 160 m, 1250 m, and 2800 m. Major phylogenetic groupings are shown on the left; the numbers at the bottom of the panel indicate incubation time in days.

Supplementary Figure 4 Dendrogram showing similarity clustering of microbial community compositions between samples from 160 m, 1250 m, and 2800 m at Stn. 8. Labelling underneath each branch indicates laminarin (La) or no-substrate (N) incubations, and the timepoint in days. Bar charts underneath the dendrogram show the relative community composition corresponding to each incubation and time point.
