# Supplementary figures and images for: Extensive Microbial Processing of Polysaccharides in the South Pacific Gyre via Selfish Uptake and Extracellular Hydrolysis
Source: Front Microbiol. 2020 Dec 18;11:583158. doi: 10.3389/fmicb.2020.583158 (PMC7775370; doi:10.3389/fmicb.2020.583158)

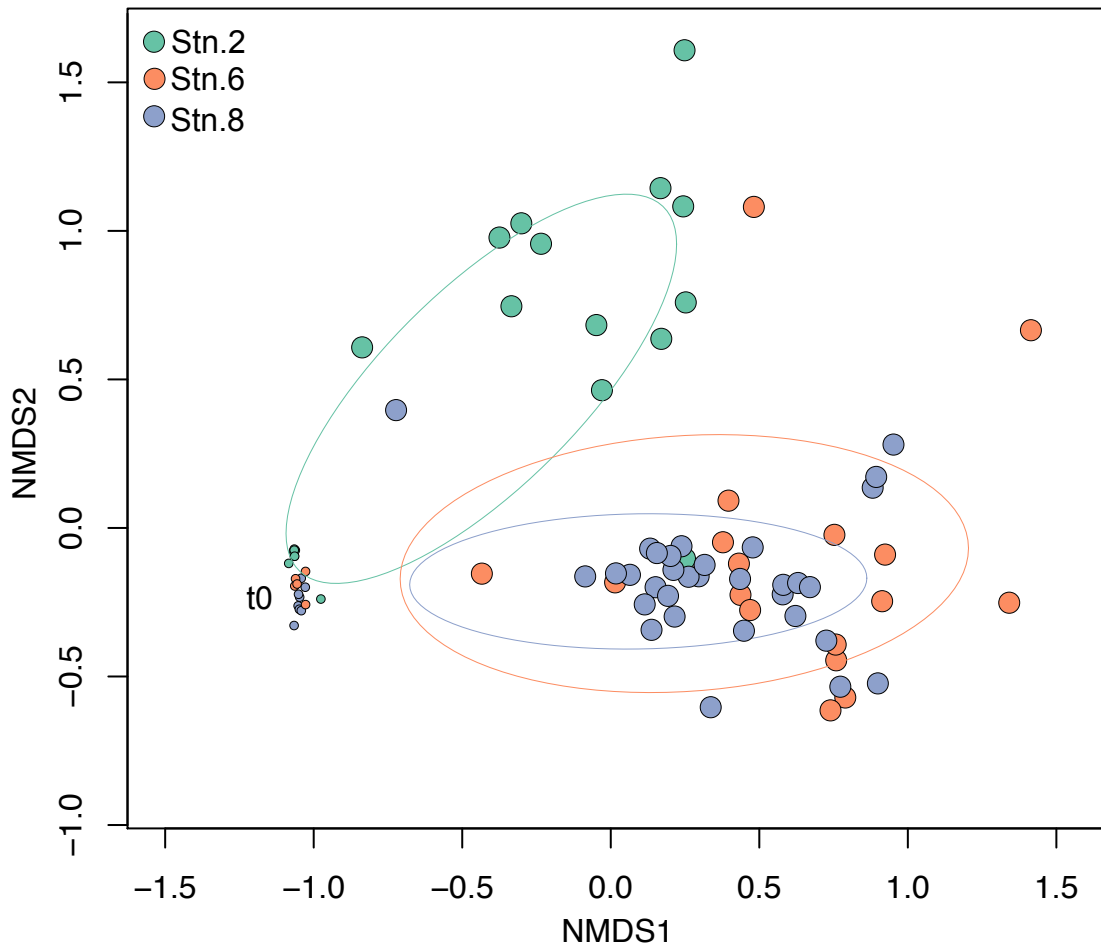

Supplement: Supplementary Figure 2 — NMDS ordination showing Bray-Curtis dissimilarity in bacterial community composition during time course incubations from Stns. 2, 6, and 8. Ovals indicate standard deviation ellipse with a confidence limit of 0.5. Initial community composition (marked t0 and shown in smaller symbols) groups together; with time, the Stn. 2 incubations become more distinct from the Stn. 6 and 8 incubations. [file Image_2.pdf]

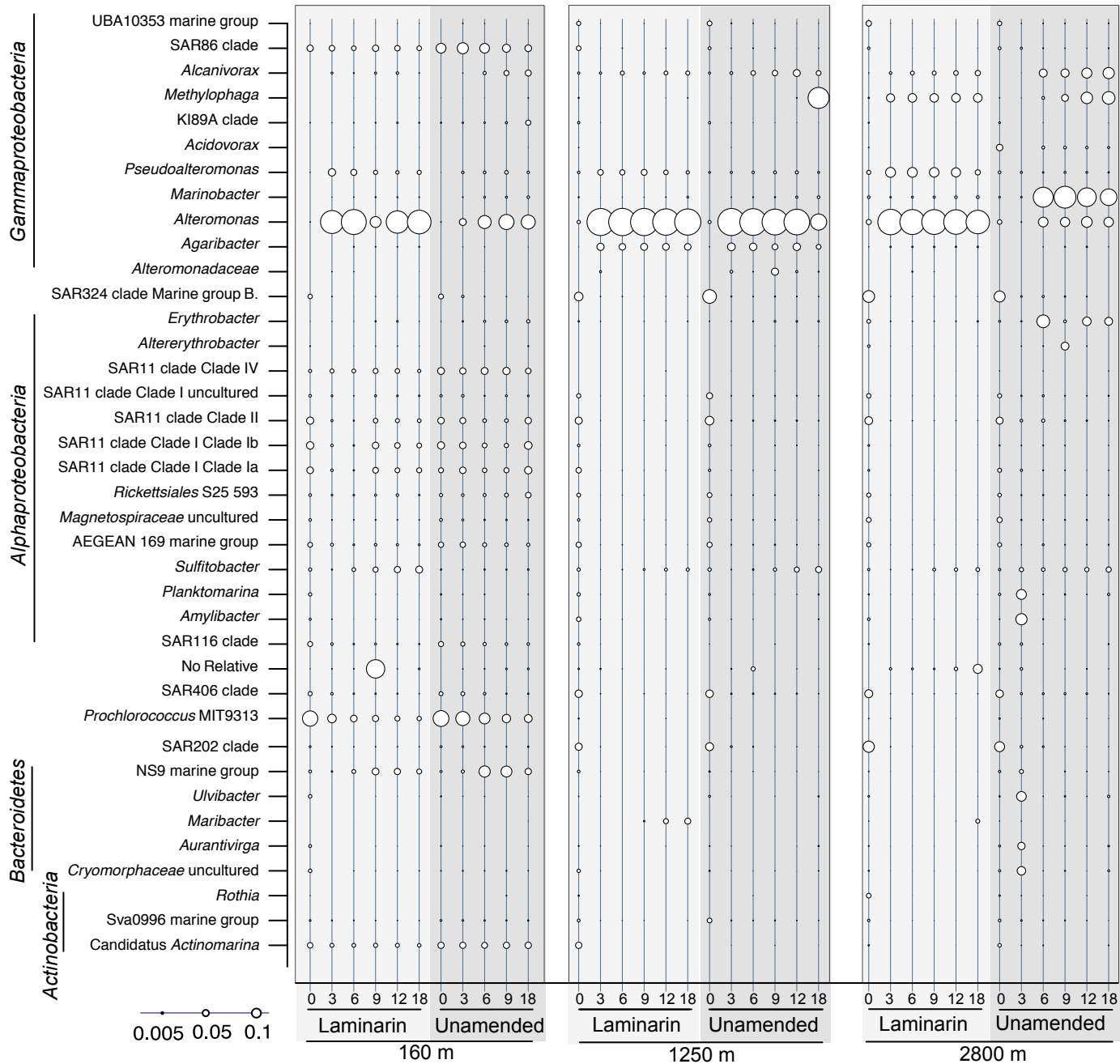

Reintjes et al. 2020 Sup. Fig. 3

Supplement: Supplementary Figure 3 — Bubble plot of bacterial genera with a minimum normalized read abundance of 0.5% for all incubations and timepoints from Stn. 8 with water from depths of 160 m, 1250 m, and 2800 m. Major phylogenetic groupings are shown on the left; the numbers at the bottom of the panel indicate incubation time in days. [file Image_3.pdf]

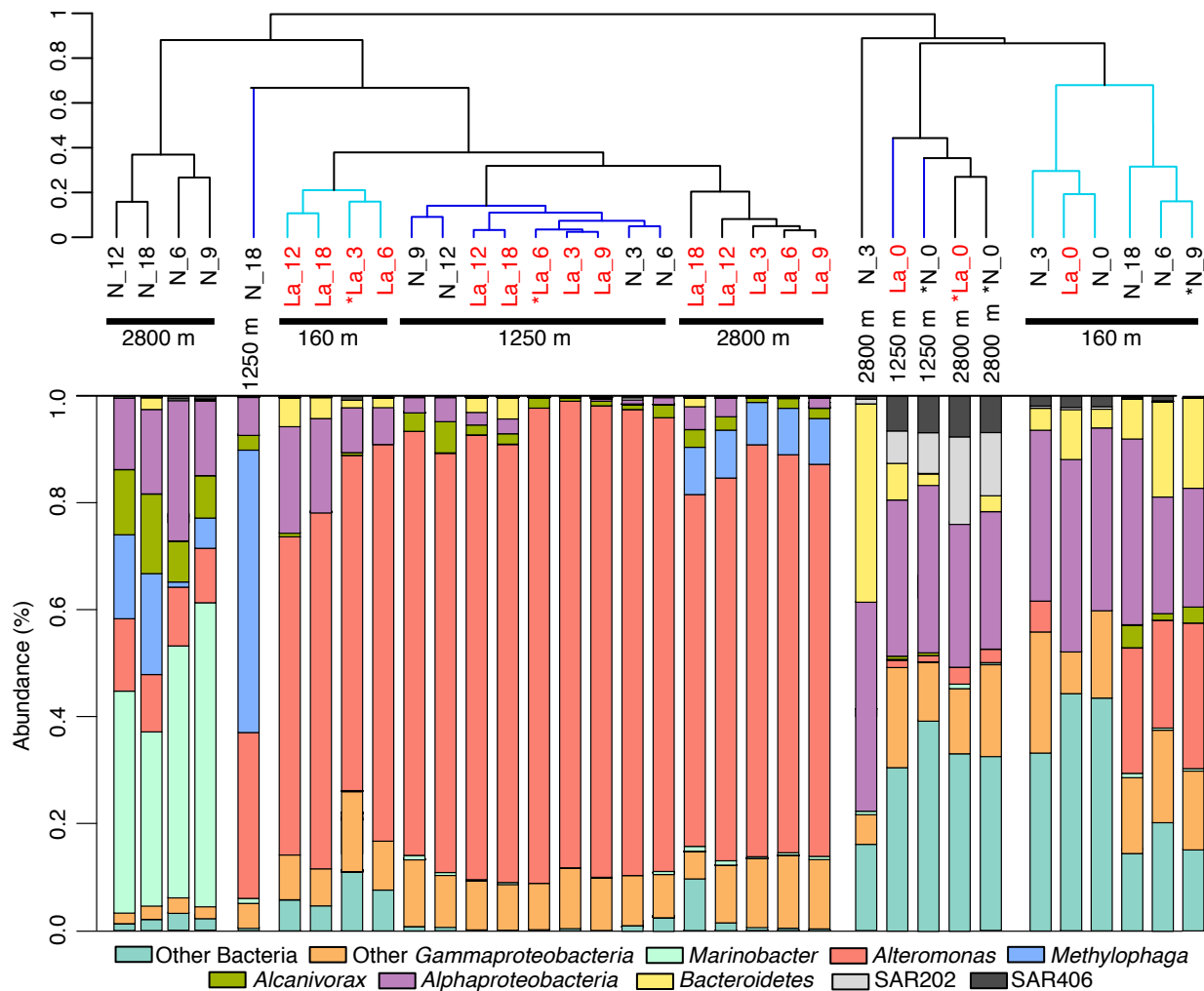

Supplement: Supplementary Figure 4 — Dendrogram showing similarity clustering of microbial community compositions between samples from 160 m, 1250 m, and 2800 m at Stn. 8. Labelling underneath each branch indicates laminarin (La) or no-substrate (N) incubations, and the timepoint in days. Bar charts underneath the dendrogram show the relative community composition corresponding to each incubation and time point. [file Image_4.pdf]
