# Supplementary material for: Extensive Microbial Processing of Polysaccharides in the South Pacific Gyre via Selfish Uptake and Extracellular Hydrolysis
Source: Front Microbiol. 2020 Dec 18;11:583158. doi: 10.3389/fmicb.2020.583158 (PMC7775370; doi:10.3389/fmicb.2020.583158)
Supplement: Supplementary Figure 1 — Bubble plot of bacterial genera with a minimum normalized read abundance of 0.5%, in FLA-PS incubations and no-substrate incubation from Stns. 8, 6, and 2. Major phylogenetic groupings are shown on the left; bubble size corresponds to relative abundance in an individual FLA-PS incubation at each timepoint. L = laminarin, X = xylan, C = chondroitin sulfate, A = arabinogalactan, F = fucoidan, P = pullulan, N = unamended incubation. Small numbers at the bottom of the panel indicate incubation time in days (0 to 18 days). [file Image_1.pdf]

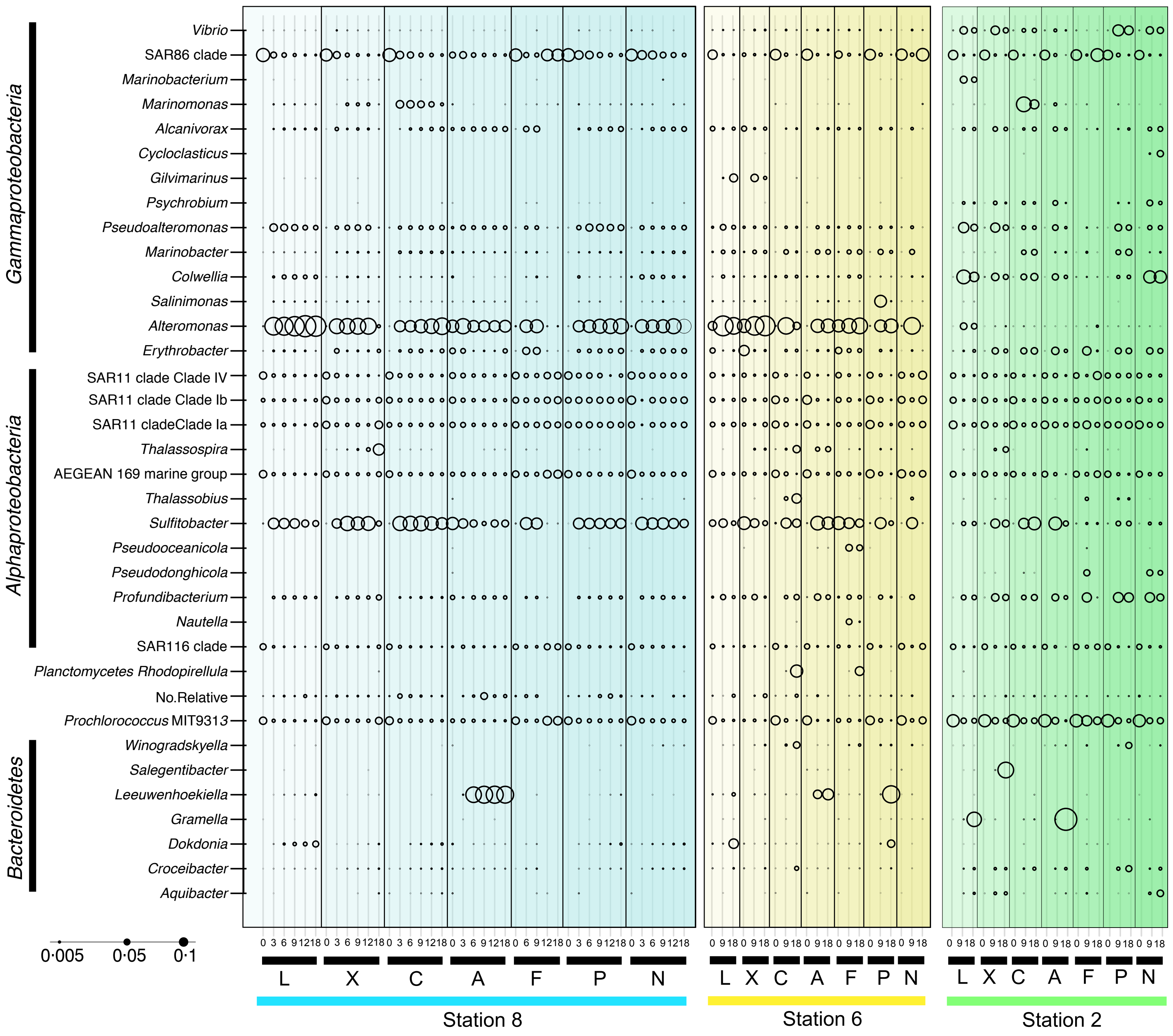

Reintjes et al. 2020 Sup. Fig. 1
